# Photopolarization of *Fucus* zygotes is determined by time sensitive vectorial addition of environmental cues during axis amplification

**DOI:** 10.3389/fpls.2015.00026

**Published:** 2015-02-03

**Authors:** Kenny A. Bogaert, Tom Beeckman, Olivier De Clerck

**Affiliations:** ^1^Research Group Phycology, Biology Department, Ghent UniversityGhent, Belgium; ^2^Department of Plant Biotechnology and Bioinformatics, Ghent UniversityGhent, Belgium; ^3^Department of Plant Systems Biology, Vlaams Instituut voor BiotechnologieGhent, Belgium

**Keywords:** *Fucus*, polarization, asymmetrical cell division, positional information, Brown algae, intrinsic factors, embryogenesis, patterning

## Abstract

Fucoid zygotes have been extensively used to study cell polarization and asymmetrical cell division. Fertilized eggs are responsive to different environmental cues (e.g., light, gravity) for a long period before the polarity is fixed and the cells germinate accordingly. First, it is commonly believed that the direction and sense of the polarization vector are established simultaneously as indicated by the formation of an F-actin patch. Secondly, upon reorientation of the zygote, a new polar gradient is formed and it is assumed that the position of the future rhizoid pole is only influenced by the latter. Here we tested these two hypotheses investigating photopolarization in *Fucus* zygotes by reorienting zygotes 90° relative to a unilateral light source at different time points during the first cell cycle. We conclude that fixation of direction and sense of the polarization vector is indeed established simultaneously. However, the experiments yielded a distribution of polarization axes that cannot be explained if only the last environmental cue is supposed to determine the polarization axis. We conclude that our observations, together with published findings, can only be explained by assuming imprinting of the different polarization vectors and their integration as a vectorial sum at the moment of axis fixation. This way cells will average different serially perceived cues resulting in a polarization vector representative of the dynamic intertidal environment, instead of betting exclusively on the perceived vector at the moment of axis fixation.

## Introduction

Patterning of an embryo is often dependent on maternally determined polarity. In most organisms at least one axis is established during oogenesis while the cell is still enclosed in parental tissue. For example, the apical-basal pattern of egg cells in land plants is maternally determined (Ueda and Laux, 2012). Fucoid zygotes are exceptions to this general pattern. The eggs are radially symmetric the moment they are released in the water column and are therefore especially interesting model systems for the study of the establishment of cell polarity (Quatrano and Shaw, 1997; Kropf et al., 1999).

Cell polarization of fucoid eggs starts directly after fertilization resulting invariably in an asymmetric cell division. The establishment of polarity is a continuum of overlapping events that are traditionally subdivided in two stages: axis selection and axis amplification (Kropf, 1997). The polarization vector is specified during the process of axis selection. The future rhizoid pole is initially specified by the sperm entry site that co-localizes with the cortical location of an F-actin site (Hable and Kropf, 2000). This axis serves as the default pathway for polarization of zygotes cultured in the absence of environmental signals. Subsequently, the zygote develops an extracellular adhesive matrix and attaches to the substrate (Vreeland et al., 1993). At this stage, environmental cues can still override the weaker default axis provided by the sperm entry site. Unidirectional light, for example, induces the rhizoids to grow from the shaded side (Hurd, 1920). The F-actin patch at the sperm entry site disassembles and a new F-actin patch is formed at the nascent rhizoid pole in accordance with the new vector (Alessa and Kropf, 1999; Kropf et al., 1999). After axis selection, there is a period of axis amplification involving localized secretion. The endomembrane system, organized by the F-actin and microtubular cytoskeleton, accumulates adhesive material preferentially at the future rhizoid pole, determining an intracellular polar axis that will be termed here the “axis amplification vector” (Vreeland et al., 1993; Hadley et al., 2006; Hable et al., 2008; Peters and Kropf, 2010). The onset of this stage is likely controlled by a developmental clock as it starts a couple of hours after fertilization (AF) (Alessa and Kropf, 1999), probably in order to avoid axis amplification before the zygote has had the chance to settle using the freshly acquired adhesive layer.

Although in intertidal habitats environmental cues are likely to be much more diverse as well as dynamic, unilateral light is the most commonly used cue for *in vitro* polarization experiments. Zygotes are plated in petridishes or on coverslips and illuminated laterally throughout the cell cycle. In the intertidal, a number of different vectors are perceived at once and these signals are integrated together (Hable, 2014, this issue). Until shortly before germination the axis remains, surprisingly, labile and susceptible to realignment to a new vector (Alessa and Kropf, 1999). The axis becomes fixed as a consequence of the local secretion of Golgi-derived material including sulfated fucan (F2) into the cell wall (Hogsett and Quatrano, 1978; Shaw and Quatrano, 1996b) and the establishment of the “axis stabilizing complex” (Fowler and Quatrano, 1995; Belanger and Quatrano, 2000). In case the environmental conditions change and a new environmental vector is perceived during the photoresponsive period, a new rhizoid site will be selected according to this new vector and amplified (Kropf et al., 1999). It is only prior to germination that the polarization axis becomes permanently fixed (Fowler and Quatrano, 1995; Belanger and Quatrano, 2000).

The new axis amplification vector is not established by mere rotation of the old one but is established *de novo* because of two reasons. (i) First, it has been found that polarized light induces zygotes to develop two rhizoids at opposite poles (Jaffe, 1958). (ii) Secondly, several factors such as the F-actin patch (Alessa and Kropf, 1999), polar secretion (Schröter, 1978), the dihydropyridine receptors (Shaw and Quatrano, 1996a), ionic currents and cortical clearing (Nuccitelli, 1978) show a reoriented polar organization quickly after reorientation as predicted by the new light vector. Moreover, cells with two F-actin patches in the short time frame after the reorientation have been observed (Alessa and Kropf, 1999).

It is assumed that the axis stabilizing vector is the fixed form of the last axis amplification vector and therefore the future rhizoid pole is identical to the new shaded hemisphere. Despite some remnants of the first environmental vector such as the polar adhesive (Schröter, 1978), the first light vector is thought to have no influence on the polarization axis. Interestingly, the new axis is assembled and amplified rapidly as it does not need additional time (Kropf et al., 1999). To our knowledge, there is no evidence for the assumed link between the *de novo* established amplification vector and the axis stabilization vector. Most reorientation experiments reorient only at one time point at the beginning of the photoresponsive period, leaving only a very short time for the initial amplification vector to leave a putative detectable influence on the final polarization axis. Secondly, the reorientation experiments use 180° changes. Cells that grow a rhizoid according to the first light vector can be either interpreted as being fixed before reorientation or having a larger influence of the first light cue than the second. Therefore, these experiments cannot exclude the possibility that the old axis amplification vector influences the final axis stabilization vector. Only Schröter (1978) used a ca. 125° reorientation at the beginning of the photoresponsive period, but reported detailed results of a single zygote only.

When the zygotes are reoriented in relation to unilateral light the axis amplification vector changes. The orientation of the polarization axis and the position of the rhizoid pole are implicitly assumed to change in one step. The observation of negative photopolarization (with the zygote developing the rhizoid at the lighted side instead of the dark side) under treatments that alter intracellular Ca^2+^ gradients (Robinson, 1996), may suggest that fixation of direction and sense involves two separate steps in fucoid cell polarization (Fowler and Quatrano, 1997). However, this has never been tested due to the complete reliance on 180° reorientation experiments (Cove, 2000). Such reorientation changes only the sense of the incoming light vector and not its direction, while reorientations with 90° change both sense and direction of environmental vector and therefore make it possible to test whether sense and direction become fixed with the same kinetics. Here we tested two implicit assumptions: (i) that the polarization process cannot be separated on a temporal scale into a two step process (ii) that the last ‘axis amplifying vector’ is identical to the ‘axis stabilizing vector’. Our results indicate that it is indeed impossible to separate the alignment of the direction and sense of the polarization vector into a two-step process. Our 90° reorientation experiments, however, contradict the hypothesis that only the last axis amplifying vector defines the axis stabilizing vector (and polarization axis), thereby offering some novel insights to longstanding views regarding *Fucus* polarity establishment.

## Methods and materials

### Culture

**Figure 1:**
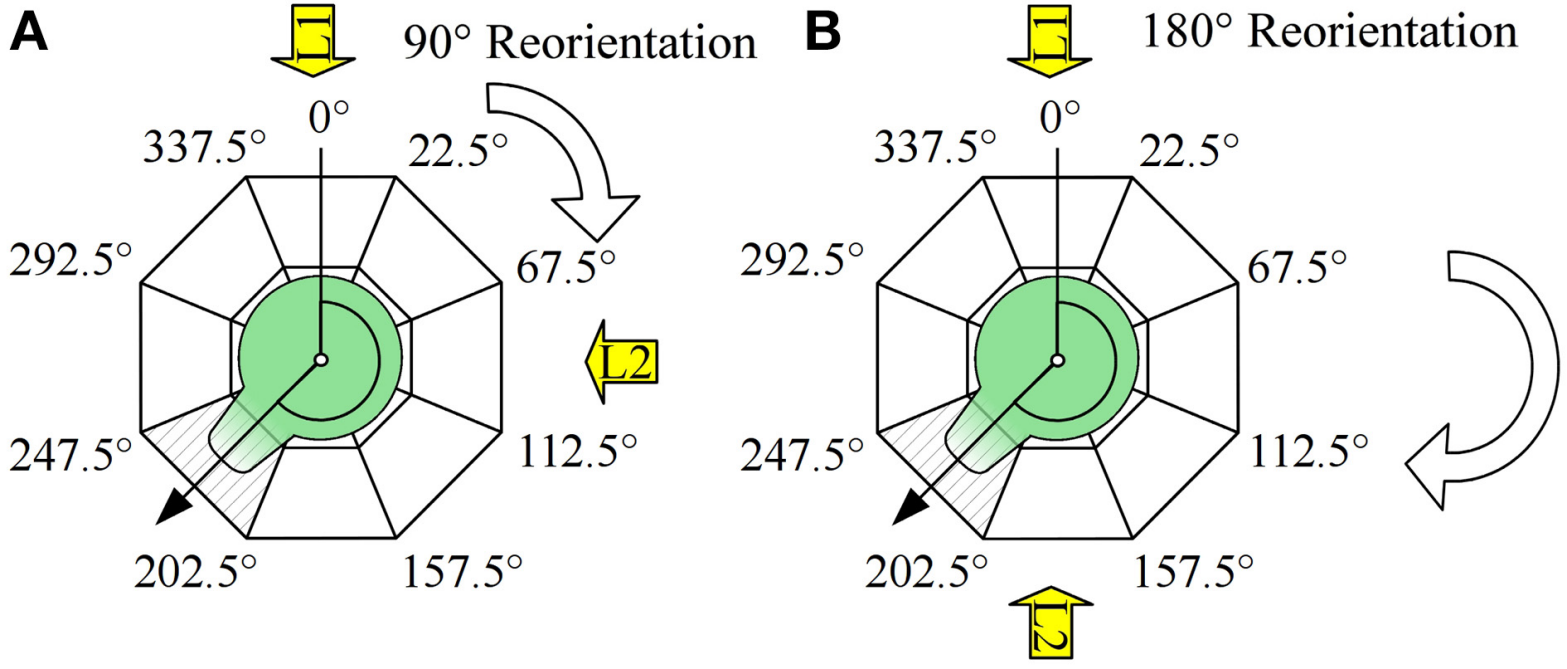
**Experimental design and scoring method and (A) 90° reorientation, (B) 180° reorientations**. Angle between the vector pointing toward the first direction of the light (black line at 0°) and the polarization vector (arrow, determined by the rhizoid outgrowth and the center of the cell) was scored by estimating its orientation on a angular scale with a 45° resolution as depicted (dashed interval). Numbers depict borders of the intervals.

Sexually mature receptacles of the fucoid alga *Fucus spiralis* Linnaeus were collected near Wimereux (France), Oostende (Belgium) and Blankenberge (Belgium) and stored at 4°C until use. Release was induced by rinsing the receptacles with tap water and subsequently placing them in natural daylight at room temperature in natural filtered seawater. The time of fertilization was considered at 1 h after exposure to daylight. Debris was filtered out using a 100 μm nylon mesh. Zygotes were plated on poly-L-coated coverslips and grown at ca. 16°C. For reorientation experiments, coverslips were carefully placed on petridishes (2–3 replicate coverslips per petridish) at 3 h after fertilization. Petridishes were exposed to cool white unidirectional fluorescent light at ca. 60 μmol photons m^−2^ s^−1^ on black sheets of paper to avoid reflection. For each replicate experiment a 90° or 180° reorientation of one petridish was executed at time points varying between 7 and 21 h AF. Each petridish underwent exactly one reorientation event. 48 h after fertilization, the orientation of the polarization vector was determined by scoring the zygotes with rhizoids in 45° intervals with the direction of the light vectors in the middle of an interval as illustrated in Figure 1. The first light vector illuminated the zygotes at an arbitrarily chosen angle of 0°, the second at an angle of 90° or 180°.

### TBO staining

Polarization of *Fucus* zygotes was assayed by Toluidine Blue O staining (TBO), which stains sulfated fucoidin indicative of polar secretion of Golgi-derived material into the cell wall (Quatrano and Crayton, 1973; Quatrano and Shaw, 1997). *Fucus* zygotes were stained for 15 min with 0.1% Toluidine Blue O ASW at pH 1.5. Slides were rinsed in 99% ethanol three times for ca. 5 min and once for 1 h before being mounted in tap water.

### Statistics

Sample sizes for each replicate experiment are listed in Supplementary Tables 1–3 together with the raw data. Second order mean angles of the polarization vector for the three samples under 90° degrees were calculated per replicate assuming a unimodal circular distribution using the procedure of Batschelet (1978). As a measure of angular concentration the *r*-value (Batschelet, 1972) was used. Significance of the mean angles was established by a testing procedure due to Hotelling (Hotelling, 1931). Parameters of a gompertz sigmoid functions were fitted through the mean values using R (version 3.1.0) and the package *nls* (Non-linear Least Squares).

## Results

**Figure 2:**
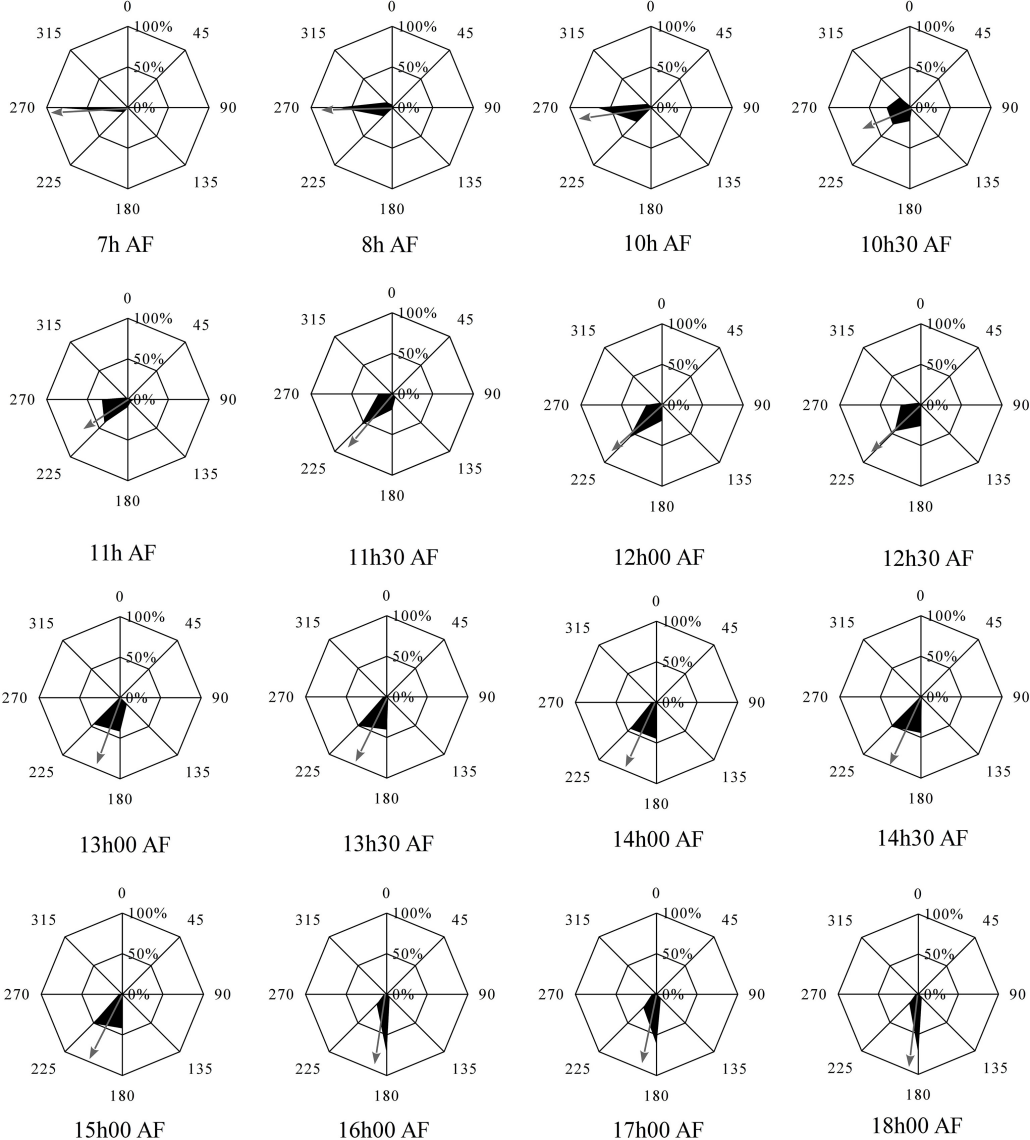
**Influence of 90° reorientation experiments on the direction and sense of polarization vector**. Second order mean of the direction and sense of the polarization vector after 90° reorientation is depicted by an arrow. Length of the arrow represents the r measure of the second order mean angle. Percentages represent the fraction of the population that shows a particular angular orientation of rhizoid outgrowth relative to the center of the cell. Angles represent the relative orientation of the rhizoid relative to the orientation of the first unidirectional light source. All mean angles of polarization vectors, including sense information, were statistically significant (df = 3, *P* < 0.05).

*Fucus* zygotes were reoriented 90° in relation to the unidirectional light source in a clockwise direction. Angles of the polarization vector with the light source (L1) were scored and represented as radial graphs for each time point of reorientation (Figure 2). Mean angles and a measure of concentration are depicted as the angle described by and the length of the arrow, respectively. All mean angles of the polarization vector were statistically significant (Hotelling test, *P* < 0.05).

**Figure 3:**
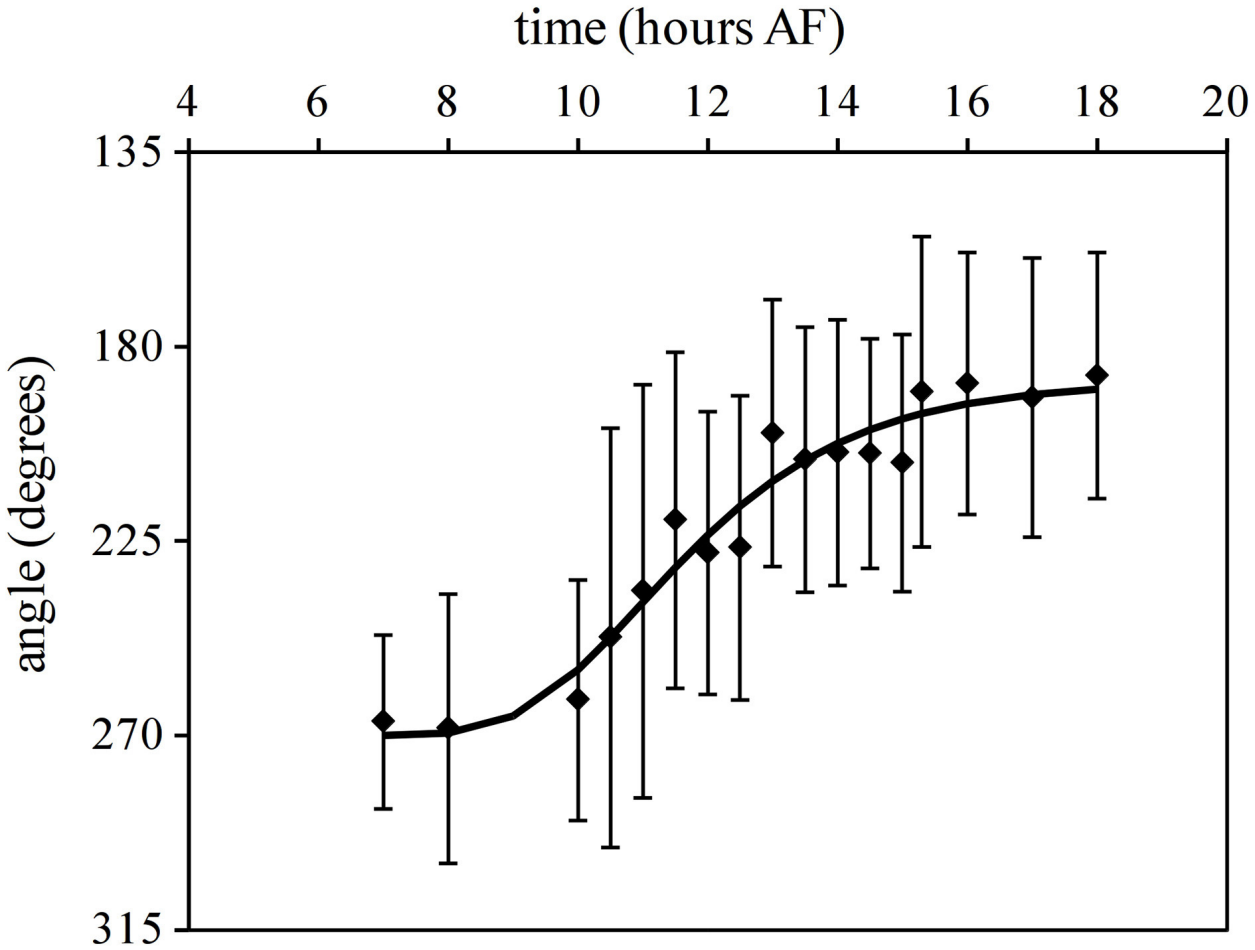
**Influence of reorientation on the fixation of direction and sense of polarization vector**. Second order mean angles for the polarization vector after 90° reorientation. Upper and lower error bars are Mardia's circular standard deviation for the mean angles (*n* > 200 in three replicate experiments). The line depicts the regressed Gompertz sigmoid that best describes the data.

The mean angle of polarization decreases gradually from 270° to 180° with increased illumination time of L1 (Figure 3). For example with the reorientation carried out at 7 h AF almost all zygotes fixed their polarization vector in accordance with L2, while a reorientation at 18 h AF produced a population of rhizoids pointing in the direction and sense described by the L1 environmental vector. During intermediate time points no enrichment of zygotes with polarization direction aligned to L1 but with misaligned sense of polarity (relative to L1) can be observed. Second order mean angles were plotted over time together with a regressed Gompertz sigmoid function (Figure 3). The angle gradually turns from an angle that is in accordance with the second light vector (270°) to one in accordance with the first light vector (180°) with increasing illumination time by the first light vector. Twelve hours AF an intermediate angle of 225° is obtained and at 15 h 30 AF, the angle is very close to 180°.

**Figure 4:**
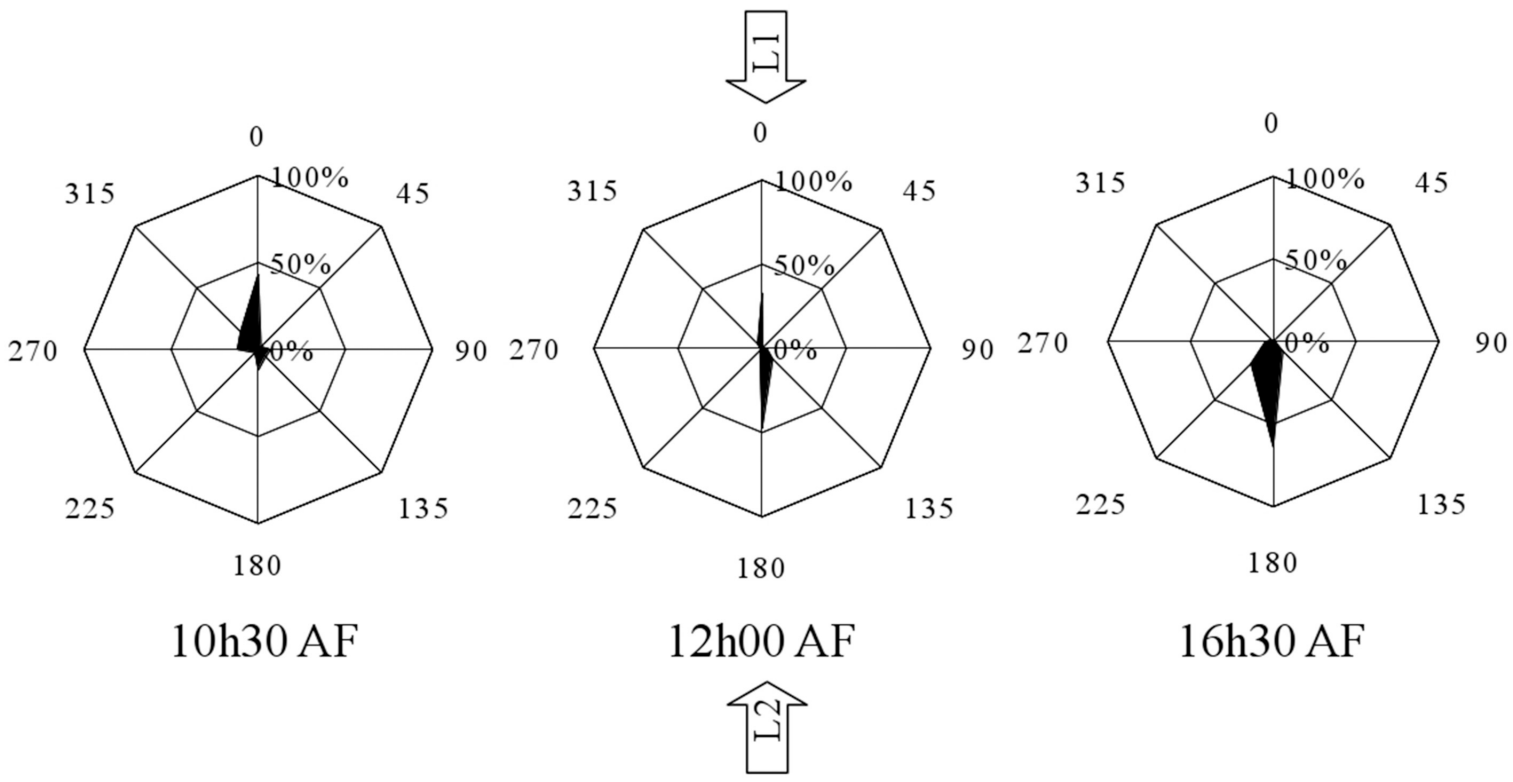
**Influence of 180° reorientation experiment on the polarization vector for the time points 10.5, 12, and 16.5 h AF**. Percentages aligned vertically represent the percentages of the population that show a particular angular orientation of rhizoid outgrowth relative to the center of the cell. Angles represent the relative orientation of the rhizoid relative to the orientation of the first unidirectional light source (*n* = 300 in three replicate experiments).

**Figure 5:**
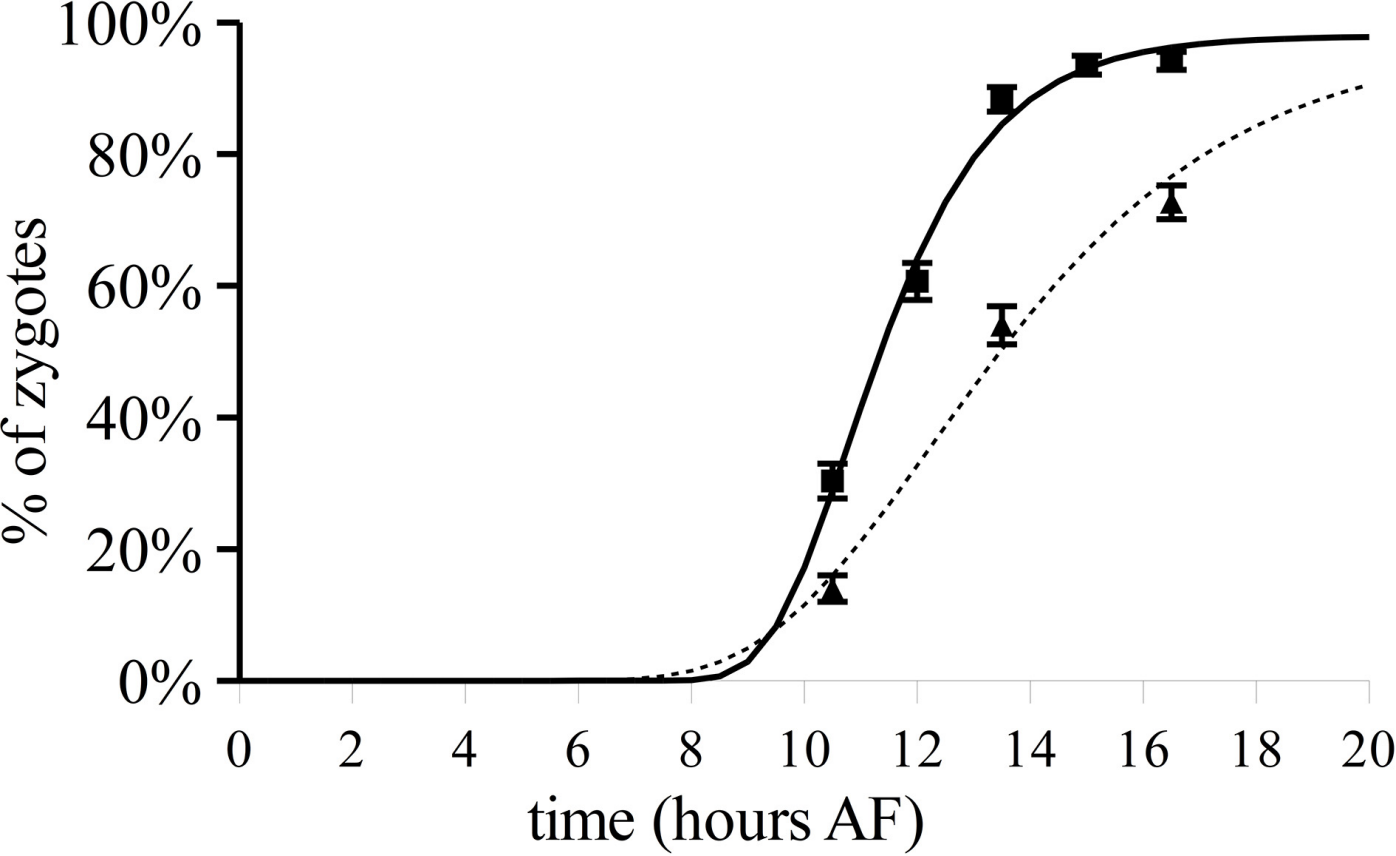
**Polarization after varying 180° reorientation over time and TBO staining in zygotes developing under unchanged unilateral light**. Fraction of zygotes having a polarization vector that points toward the shaded side in relation to L1 (squares). Percentage of zygotes staining asymmetrically with TBO under unilateral light (triangles). Upper and lower error bars are standard deviations (*n* = 300 in three replicate experiments). The lines depict the regressed Gompertz sigmoids that best describes the data (continuous, photopolarization according to L1; dotted, TBO patch staining).

In a separate experiment *Fucus* zygotes were reoriented 180° at 10.5, 12, 13, 14.5, and 16.5 h AF. One hundred and eighty degrees reorientations at 10.5 h and 12 h AF produced a diametrically bimodal circular distribution (Figure 4). For example, at an intermediate time point of a reorientation at 12 h AF, 33 ± 2.71% of the zygotes polarized in the interval determined by L2, 47.67 ± 2.88% polarized in the interval determined by L1, while only 19.33 ± 2.28% polarize in one of the other six intervals (mean ± standard deviation) (Figure 4). The percentage of zygotes that fix a polarization vector on their shaded side in respect of L1 is plotted over time (Figure 5, squares), which results in a very similar curve like the one that describes the reorientation of the angle at 90°. Under 180° reorientation 50% of zygotes are polarized according to L1 at about 11.5 h AF, while a 90° reorientation results in a polarization axis of 45° with respect to L1 and L2 around the same time point.

To determine the timing of the establishment of the axis stabilizing vector the polarized secretion of Golgi-derived material was monitored in populations of zygotes grown in unilateral light using TBO staining as a marker. The percentage of zygotes with asymmetric deposition of F2 fucoidin as assayed by TBO staining is plotted over time (Figure 5, triangles, dotted line) and reaches ca. 50% only at about 13.5 h AF.

## Discussion

Currently it is accepted that *Fucus* zygotes in the process of polarization commit to the last applied environmental vector before axis fixation (Bisgrove, 2007; Hable and Hart, 2010). Our data cannot be explained by the currently accepted scenario. Different possible scenarios for the resultant rhizoid orientation are detailed below.

**Figure 6:**
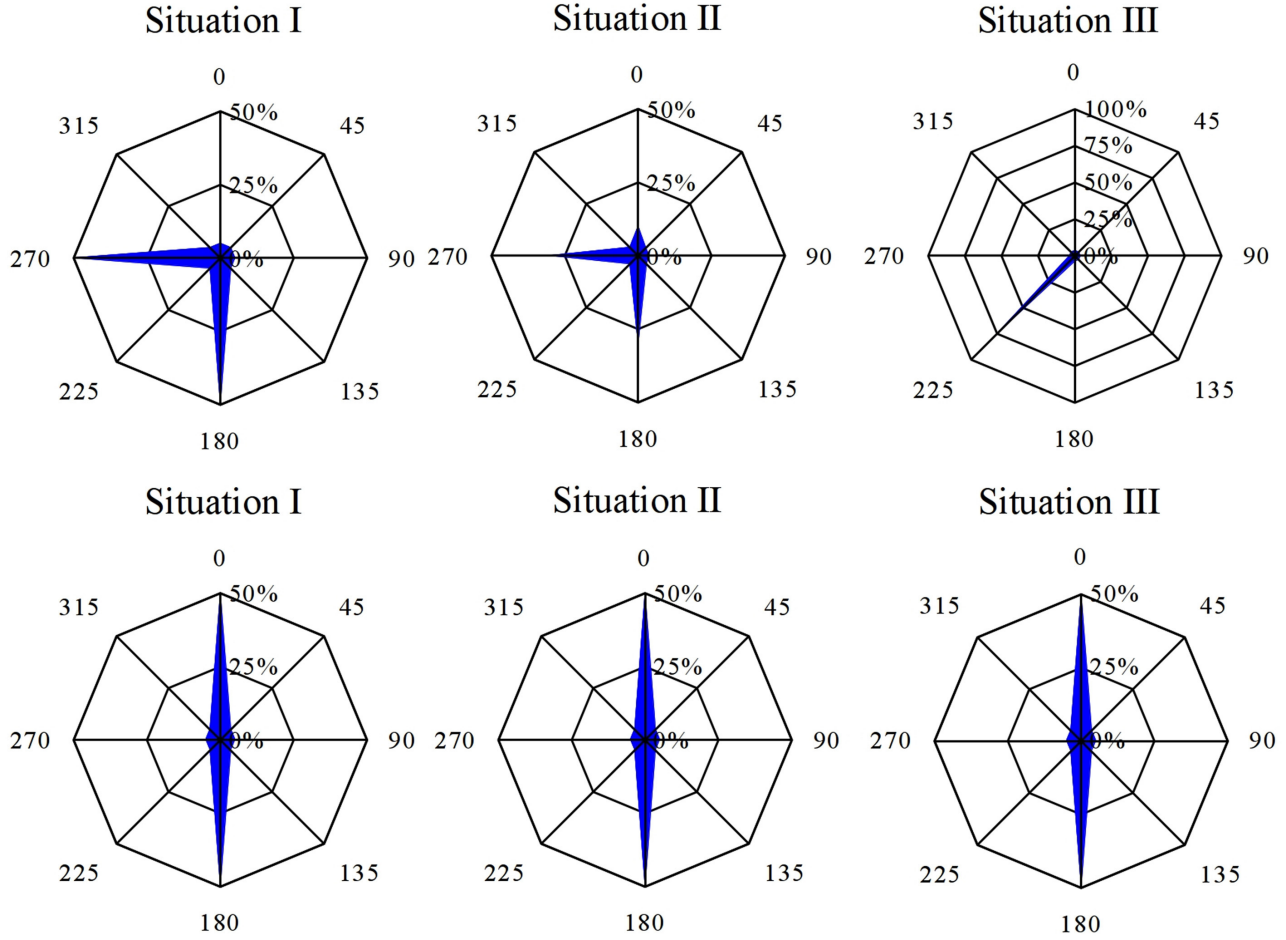
**Resultant angular distribution for each of the different polarization scenarios considered**. Note that 180° reorientation yields for each of these situations a diametrically bimodal distribution (see text for explanation).

Under the currently accepted model, whereby reorientation of polarizing zygotes prior to axis fixation results in *de novo* establishment of a new axis amplification vector (Figure 6, Situation I), we can expect a mixture of embryos with rhizoids fixed according L1 or L2 light vector. A 90° reorientation would result in a bimodal distribution of zygotes polarized at an angle 90° relative to each other. Under a 180° reorientation these two groups will be diametrically opposed and result in a diametrically bimodal distribution.

An alternative hypothesis, originally proposed by Cove (2000) and Fowler and Quatrano (1997), requires a decoupling of both the orientation, defined as the axis along which a zygote polarizes, and the sense of the polarizing vector, the position where the rhizoid is formed. This hypothesis would result in a fraction of zygotes with negative photopolarization, i.e., zygotes that polarize toward the illuminated instead of the shaded side relative to the first light cue (Figure 1, Situation II). As an example, if the fixation of the sense of the polarization vector lags behind the fixation of its orientation we expect a fraction of zygotes to have aligned both sense and direction to the L1 light vector, however another fraction that fixed the direction only according to L1 will find itself confused in the situation of L2 illumination. Here we expect about half will eventually polarize toward the L1 light source, while the other half develops its rhizoid away from the first light vector in addition to the previously mentioned fraction. When reorienting 180°, the fixation of the direction is irrelevant as it is the same for both light directions and will result again in a diametrical bimodal distribution. The fraction with negative photopolarization will not be discernible from the fraction that polarizes according to L2. At none of the time points of reorientation an over-representation of the fraction with fixed polarization direction but misaligned polarization sense (relative to the first light vector) could be observed. Our experiments exclude the possibility that the cell fixes the direction before the sense of the polarization vector.

Both scenarios imply the complete breakdown of the old axis that is “forgotten” in favor of the *de novo* assembly of a new one. In other words the last axis amplification vector is assumed to determine the axis stabilization axis, while the old axis amplification vector is disassembled and has no influence on the future rhizoid site.

Alternatively, the initial polarization vector (including F-actin patch) may be disassembled, but not without leaving a trace or “imprint” in the cell or cell wall (Figure 1, Situation III). The information provided of multiple sequential light cues will then be integrated at the time of axis fixation and result in an intermediate angle, if oriented with 90°. When reoriented 180° the light cues are diametrically opposed. Therefore, they will outweigh each other and the resultant angle will be according to the light cue that happens to be the strongest one and the distribution will be diametrically bimodal again. It is important to note that simultaneous fixation of direction and sense of the polarization vector is inherent to this scenario.

**Figure 7:**
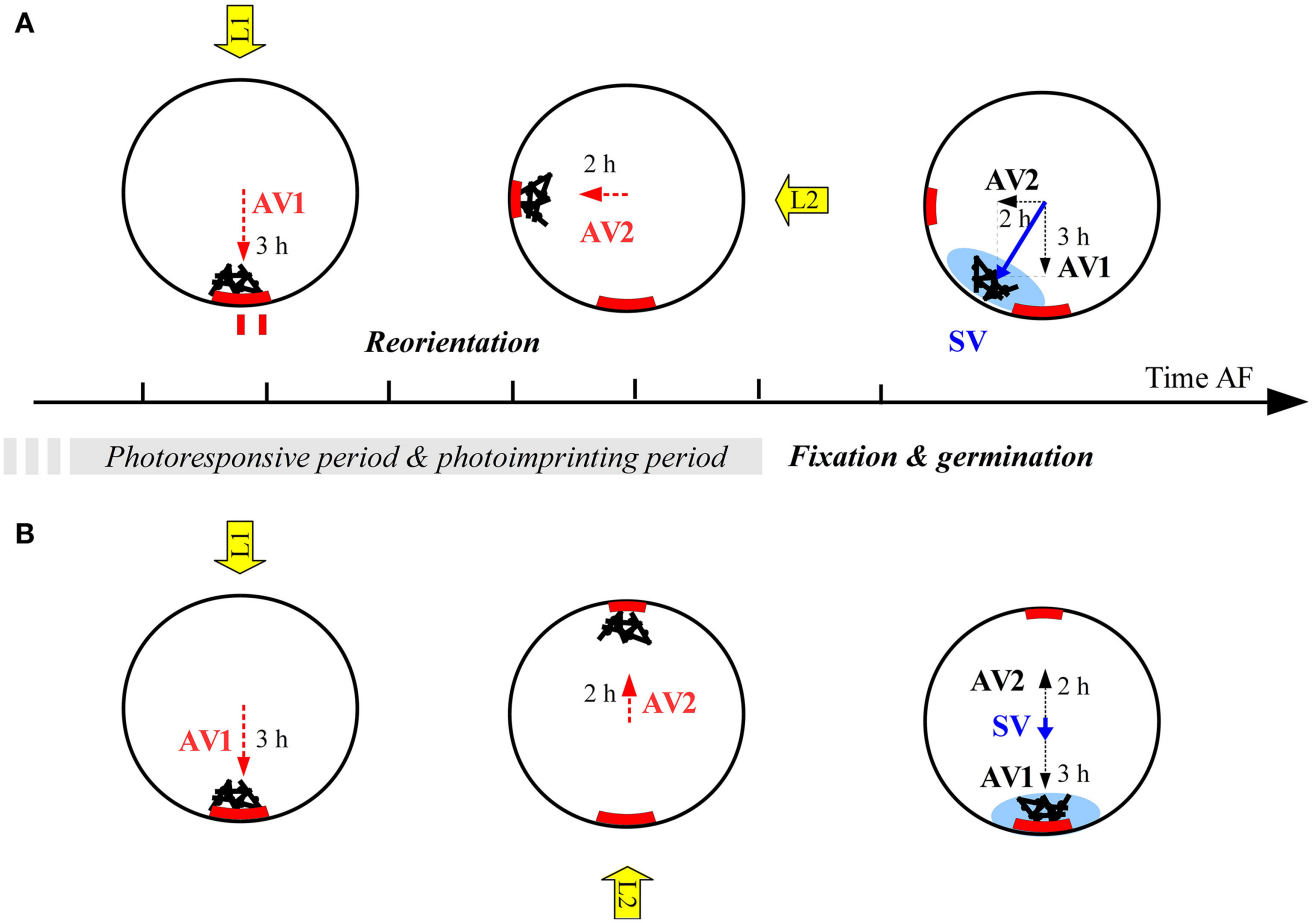
**Proposed scenario for integration of polarization vectors as vectorial sum under after reorientation of 90° (A) or 180° (B)**. AV, axis amplification vector; SV, axis stabilization vector. Red block arcs depict the hypothesized intrinsic factor responsible for the imprinting of unknown nature. Blue shading denotes F2 deposition as assayed by TBO staining. Hours denote duration of illumination under different light regimes and therefore the strength of each vector. Black bars and spheres denote the polar endomembrane system and F-actin deposition symbolizing the entire axis amplification machinery.

Indeed, we observed a different pattern than predicted by the two first scenarios. When reorienting 90°, zygotes show intermediate angles, which gradually rotate with increasing L1 illumination time until the polarization axis is completely aligned with L1. The imprints of the polarization vectors following the perpendicular light vectors L1 and L2 (in terms of asymmetrical distribution of intrinsic determinants) will be added up and result in an intermediate angle (Figure 7). The longer the exposure to L2, the stronger the asymmetrical imprint of intrinsic determinants and the larger the influence of the L2 light vector will be at the moment of fixation. The length of each axis amplification vector in Figure 7 is proportional to this influence and the length of the illumination time within a certain time frame, roughly between 9 and 14 h AF, after which the axes are integrated and fixed. This period is much likely preceded with a period during which the cells can sense a light cue but are not able to imprint it in the cell wall yet. This is suggested by the fact a large fraction of the cells are already photoresponsive at 1–3 h AF in *Fucus distichus* (Kropf et al., 1989) at which time point the cells do not have secreted a uniform adhesive layer yet. It is important to note that simultaneous fixation of direction and sense is inherent to this scenario.

Under 180° reorientation, the imprinting model implies a different interpretation of the point at which half of the cells commit to the first light vector compared to the previously discussed models. Traditionally this point is interpreted as a marker for axis fixation and denotes the point at which half of the cells have fixed their polarization axis and are therefore not sensitive any more to the second light vector (Quatrano, 1973; Kropf, 1989; Quatrano and Shaw, 1997). Under the imprinting hypothesis this is the point at which both light vectors equally influence the fixation of the polarization axis. Therefore, the imprinting model implies that the moment of axis fixation, assayed by TBO staining, occurs with slightly different kinetics compared to the commitment to the L1 vector (Figure 5). Indeed the data suggest half of the zygotes of the same three populations has an asymmetric TBO staining pattern only after 13.5 h, which indeed coincides roughly with the end of the responsive period. Two hours earlier (10 h AF) 50% of zygotes already have determined the side of rhizoid formation as determined by L1. In *Fucus distichus*, however, it has been reported the moment of 50% L1 commitment and 50% of TBO staining coincided by 10 h AF (Shaw and Quatrano, 1996a,b) which contradicts our results. In contrast to the previous analysis, we report TBO staining at more than one time point and in more than one replicate population. Particularly the increased temporal resolution in the estimation of the F2 deposition kinetics might explain the apparent contradiction.

The different interpretation of the commitment to L1 in the ‘imprinting model’ links up with the controversy whether there exists an axis fixation event that is temporally distinct from germination (Quatrano, 1973) or whether the polarization axis becomes fixed by local F2 deposition at the moment of germination itself as outlined by Robinson et al. (1999). While it is accepted that the axis is fixed before germination (Goodner and Quatrano, 1993), experiments involving an osmotic block of germination and subsequent repolarization might suggest there is no axis fixation before germination (Jaffe, 1990; Robinson, 1996). Therefore, the commitment to the first light vector prior to polar growth as observed by Quatrano (1973), should have a different explanation than axis fixation. This explanation can be provided by the proposed imprinting model. The here-presented scenario implies that axis fixation occurs later than previously assumed based on reorientation data. If somehow the fixation of the cells is postponed (e.g., by osmotic block of polar growth), the L2 light source will still be able to reverse the polarization axis (Robinson et al., 1999).

It has been pointed out that the rapid fixation of a new polarization axis after reorientation is interesting and the fact that the axis remains labile for a long time is considered surprising (Kropf et al., 1999). Indeed one may expect *a priori* that the process of amplification should occur with a constant speed. The long lability of the axis is surprising as it is difficult to imagine a distinct advantage in the intertidal. The moment the cells adhere at around 4–6 h AF one may expect a faster development would be possible and advantageous. This period of sensing the environment can be better understood in the context of the imprinting model as a period of averaging all environmental cues the cell perceives during a 5-h interval. Fucoid zygotes in the intertidal rarely undergo continuous unilateral light as the sun migrates during this interval. Instead of betting on the last perceived vector, cells seem to average the perceived vector. This results in a polarization vector much more representative of the environment to which they need to adapt their development.

In conclusion, the here presented data together with the discussed published data can best be explained by the vectorial addition of imprints made during a large fraction of the cell cycle. How the information of the first environmental cue is stored, is not known. One can only speculate on the molecular nature of the cellular memory. Because the extracellular matrix has been shown to be an important way of storing developmental information controlling the cell fate (Bogaert et al., 2013), this part of the cell is the best location to search for the postulated intrinsic factor.

While fucoid zygotes are responsive to an impressive range of informational cues and develop in a dynamic environment with cues that may change from direction, cells normally develop only one rhizoid suggesting that multiple cues are integrated to generate a single one. While the axis amplification on itself is relatively well-documented, the way these cues are integrated is only circumstantially known (Hable and Hart, 2010). We have provided a better understanding in the way zygotes integrate sequentially applied vectors. How zygotes respond to simultaneously applied cues is still poorly understood. All cues that induce a signal are believed to be integrated in the common pathway upstream of establishment of the axis amplification (Kropf et al., 1999). But at which point their signaling cascade converges with each others ones is unknown. One may propose the here proposed imprinting mechanism would provide an alternative mechanism. But the fact that cells have only one amplification vector at the same time suggests this simultaneous integration occurs at the level of polarity signaling as proposed by Kropf et al. (1999).

### Conflict of interest statement

The authors declare that the research was conducted in the absence of any commercial or financial relationships that could be construed as a potential conflict of interest.
